# Supplementary material for: Rotating edge-field driven processing of chiral spin textures in racetrack devices
Source: Sci Rep. 2020 Nov 23;10:20400. doi: 10.1038/s41598-020-77337-y (PMC7684311; doi:10.1038/s41598-020-77337-y)
Supplement: Supplementary file 1 — Supplementary Information. [file 41598_2020_77337_MOESM1_ESM.pdf]

Supplementary material: Rotating edge-field driven processing of chiral spin textures in racetrack devices

Alexander F. Schäffer,[1,2] Pia Siegl,[3] Martin Stier,[3] Thore Posske,[3] Jamal Berakdar,[1] Michael Thorwart,[3] Roland Wiesendanger,[2] and Elena Y. Vedmedenko[2]

[1]*Institute of Physics, Martin-Luther-Universität Halle-Wittenberg, D-06120 Halle (Saale), Germany*

[2]*Department of Physics, Universität Hamburg, D-20355 Hamburg, Germany*

[3]*I. Institute for Theoretical Physics, Universität Hamburg, D-20355 Hamburg, Germany*

A. ATOMISTIC SIMULATIONS ON A MONOLAYER WITH AN HCP(111) STACKING

For the systems, discussed in the main text, we have used a discretization into cubic simulation cells. However, ultrathin films such as the Pd/Fe bilayer on Ir(111) typically grow in hexagonal close-packed (hcp) stacking. Here, we strictly use the hcp structure of magnetic adatoms and show the applicability of the presented writing and deleting mechanism also to these cases. Additionally, the skyrmions are stabilized by a combination of the DMI and the geometric confinement entering as fixed boundary conditions. This represents another example of nanometer sized skyrmions in the absence of global magnetic fields.
For the atomistic simulations, classical Heisenberg magnetic moments $\mathbf{S_i} = (S_i{}^x, S_i{}^y, S_i{}^z)$ of unit length $\boldsymbol{\mu}_i/\mu_\mathrm{s}$ are placed at each lattice point. The energy of the system is given by the Hamiltonian

$$\mathcal{H} = -J\sum_{\langle ij\rangle}\mathbf{S}_i\cdot\mathbf{S}_j - \sum_{\langle ij\rangle}\mathbf{D}_{ij}\cdot(\mathbf{S}_i\times\mathbf{S}_j) - B_z\sum_i S_z - \sum_i \mathbf{B}_i(t)\cdot\mathbf{S}_i - K_z\sum_i (S_i^z)^2, \quad (1)$$

where $J > 0$ is the ferromagnetic exchange coupling between nearest neighbors, $\mathbf{D}_{ij}$ is the DMI vector, B_z is a global magnetic field, K_z is the perpendicular magnetic anisotropy and $\mathbf{B}_i(t)$ is a local space- and time-dependent magnetic field. The material parameters were set to $K_z = 0.05\,J$, $D = 0.07\,J$ and the magnetic moments at the edges are fixed to $\mathbf{S} = \hat{\mathbf{e}}_z$ to stabilize skyrmions in the absence of an additional external field. The spins at $x = 0$ were rotated parametrically. The time scale in these calculations can be expressed by $dt = \tau\mu_s/\gamma J$ with the reduced time step τ and the gyromagnetic ratio γ. For $\mu_s = 2\mu_B$, $J = 5.72$ meV and $\tau = 0.01$ a simulation step corresponds to the time span of $\approx 10^{-14}$ s. The frequency of rotation at the edge of the stripe was varied between 1 and 10 GHz. The propagation velocities were similar to those found in micromagnetic simulations.
The dynamics of the free spins is determined by the LLG equation

$$\dot{\mathbf{m}}_i(t) = -\frac{\gamma}{1+\alpha^2}\left[\mathbf{m}_i\times\mathbf{B}_i^{\mathrm{eff}} + \alpha\mathbf{m}_i\times\left(\mathbf{m}_i\times\mathbf{B}_i^{\mathrm{eff}}\right)\right] \ , \quad (2)$$

with the effective magnetic field

$$\mathbf{B}_i^{\mathrm{eff}} = -\frac{\partial\mathcal{H}}{\mu_s\partial\mathbf{S}_i} \ . \quad (3)$$

Fig. S1 shows a sequence of snapshots of the creation and deletion of magnetic skyrmions using atomistic LLG simulations on a triangular lattice with 8×100 spins. The excited

line of magnetic moments is visualized by the gray screw, also indicating the rotational sense. As shown in the micromagnetic simulations presented in the main text, skyrmions can be injected into the sample. In Fig. S2 the spins along the screw are rotated in opposite direction. Particularly interesting is that the rotation against the DMI chirality destroys skyrmions only on one side of the excited line as can be seen in time steps t_3 to t_6 of Fig. S2. In conclusion, this shows that skyrmions stabilized by DMI and fixed boundary conditions can be created and annihilated similar to the open systems discussed in the main text. Furthermore, the hexagonal lattice has no fundamental impact on the proposed control mechanism.

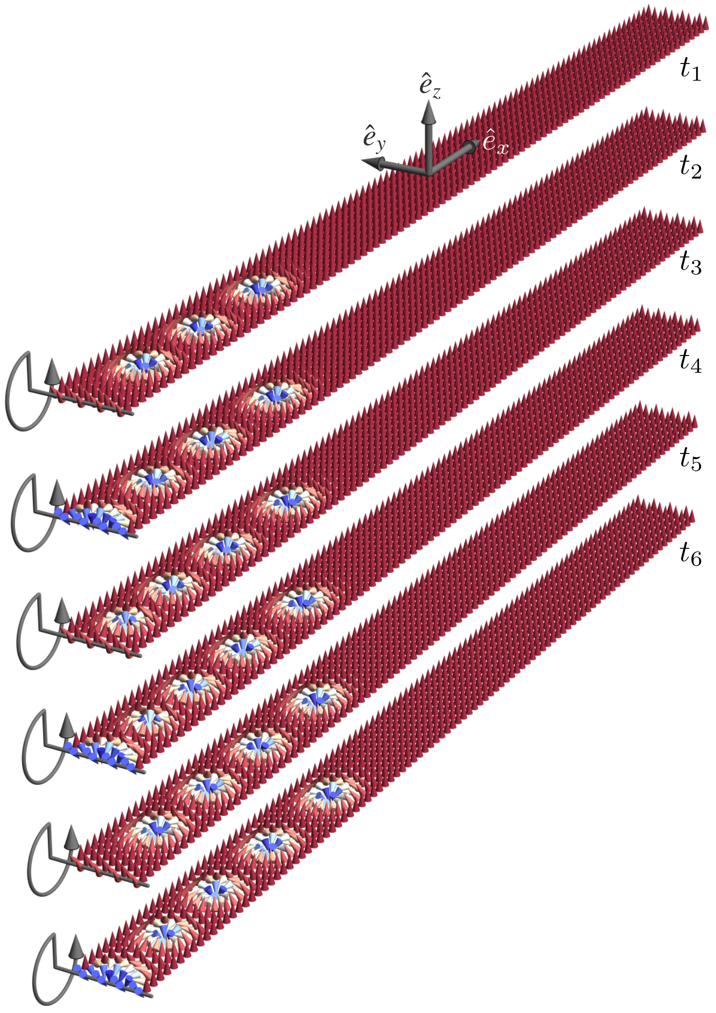

Fig. S1. Skyrmion creation in a magnetic stripe from atomistic simulations. Snapshots corresponding to the writing of individual skyrmions by a local rotating edge-field following the chirality of Dzyaloshinskii-Moriya interaction.

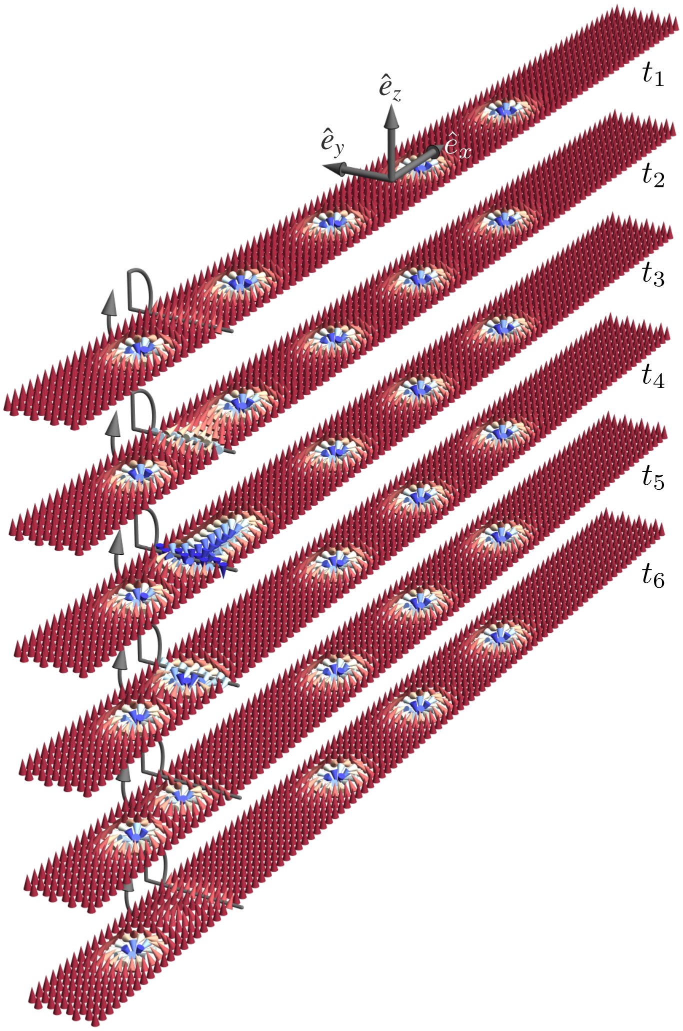

Fig. S 2. Skyrmion deletion in a magnetic stripe from atomistic simulations. Local rotating edge-field opposite to the rotational sense in Fig. S1 leads to the deletion of a skyrmion in $+x$ direction.

B. PHASE DIAGRAM OF SKYRMION CREATION FOR GAUSSIAN ROTATION

The creation processes of either domain walls or skyrmions discussed in the main paper have been modeled by a simplified rotation scheme, namely a uniform rotation of a distinct number of involved edge magnetic moments due to a rotating magnetic field. While for domain walls this intuitively is the optimal scheme, a rotation respecting the intrinsic circular form of skyrmions is a more adequate skyrmion creation process. As we will show in the following, such a tailored rotation allows for higher creation rates, which in the context of information technologies is a desirable goal.

We study the general properties of the skyrmion creation and compare different rotation schemes of a model system. To this end, we consider a square lattice of 128 x 128 lattice sites with ferromagnetic boundary conditions and system parameters of $A_{\text{exch}} = 0.69$ pJ m^{-1}, $D = 0.267$ mJ m^{-2} and $B = 0.0864$ T. Here, the ground state is the ferromagnetic state, with all magnetic moments pointing in $+z$-direction. Furthermore, we directly rotate the magnetic moments at the edge of the sample at $x = 0$ which reflects the limits of sufficiently large magnetic fields. Considering the uniform rotation of all involved boundary moments, the maximum rotation frequency resulting in a stable skyrmion depends on the number of rotated edge moments, see Fig. S3(b). An optimum is found for 20 to 60 rotated moments where a rotation with maximum frequency of $\nu = 12.5$ GHz can create a stable skyrmion.

A skyrmion creation by a uniform rotation is only possible due to the directional sense of the DMI inclining the outer moments towards the outside, such that a circular structure is formed. The creation speed is therefore determined by this process. However, it can be increased if the inclination of the moments is itself induced by the rotation. A significant improvement is achieved by a tailor-made rotation which we call the Gauss rotation. The name and rotation protocol are motivated by the z-component of the normalized magnetization m_z of the cross section of a skyrmion which can approximately be fitted by a shifted Gauss function, i.e.

$$m_z(y) = -2\exp\left[-\left(\frac{y-y_0}{\sigma}\right)^2\right] + 1. \tag{4}$$

Here σ is a measure of the width of the Gauss distribution, y refers to the position of the magnetic moment and y_0 to the center of the skyrmion, coinciding in the following with the center of the edge. Fig. S3(a) illustrates the scheme of the Gauss rotation. The moments

(black arrows) rotate around the spatially dependent rotation axis (red arrow) and are depicted in their initial state (dashed line) and after half a rotation (continuous line). The angle φ between $\mathbf{m}(t = 0)$ and $\mathbf{m}(t = \frac{T}{2})$, T being the period of the rotation, depends on the Gauss function in equation 5, namely

$$\cos(\varphi(y)) = -2\exp\left[-\left(\frac{y - y_0}{\sigma}\right)^2\right] + 1. \tag{5}$$

Using this rotation, we find the creation diagram of the Gauss rotation, see Fig. S3(c). The Gauss rotation yields the possibility of skyrmion creation at significantly higher frequencies up to 20.8 GHz, i.e., a creation of a skyrmion within 50 ps, for an optimal Gauss width $\sigma = 20$. This underlines the increased efficiency of the rotation, if it adequately forms the skyrmion shape.

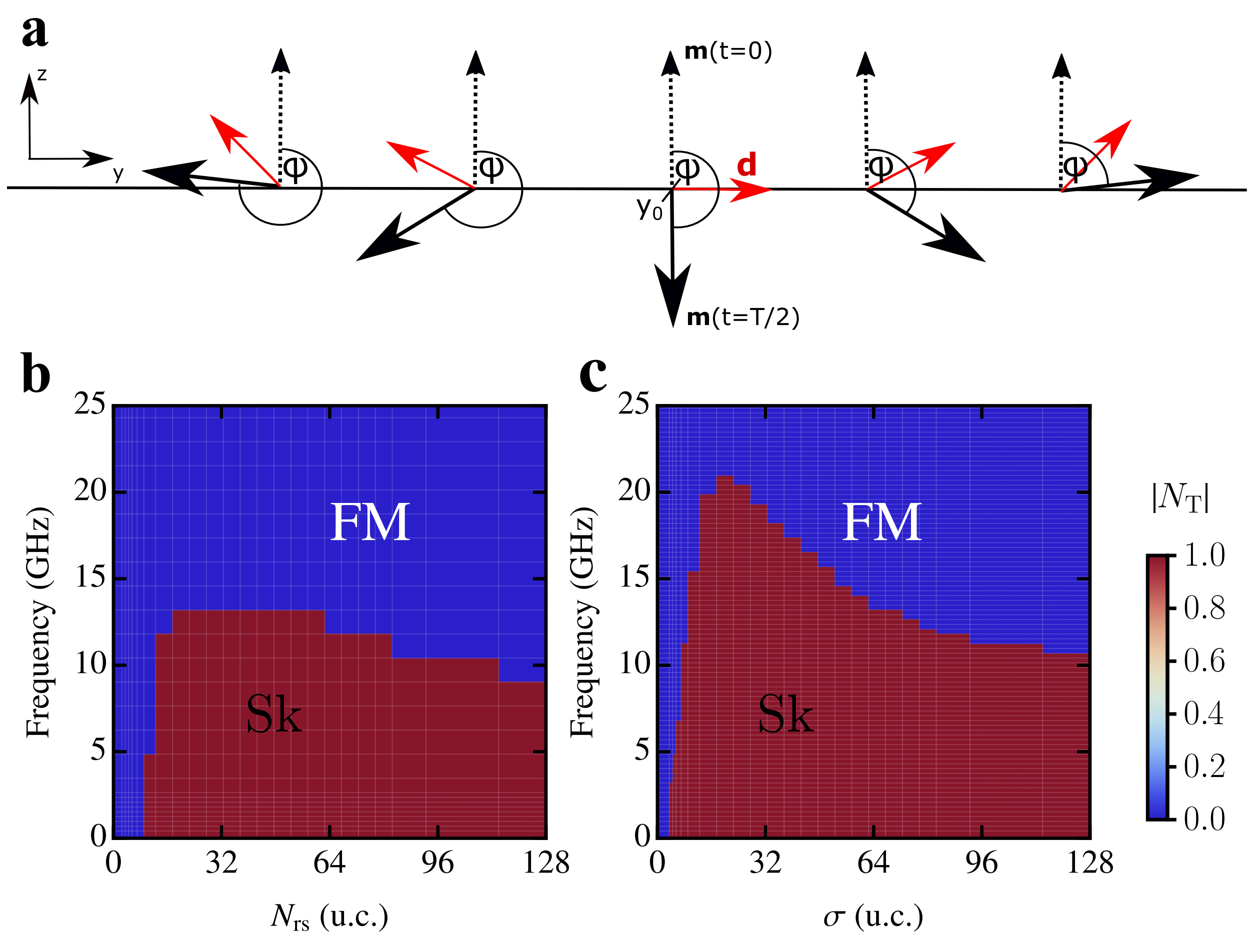

Fig. S 3. (a) Scheme of the Gauss rotation. The magnetization $\mathbf{m}$ is depicted for the initial state (black dashed arrow) and after half a rotation (black arrow) around the spatially dependent rotation axis (red arrow). (b,c) Phase diagrams of the creation of a single skyrmion by either the uniform (b) rotation or the Gauss rotation (c). Depicted is the absolute value of the topological charge in dependence of the number of the rotated magnetic moments N_{rs} (uniform rotation) or the Gaussian width σ (Gauss rotation) and the rotation frequency ν. The skyrmion is created at the end of a complete rotation, but as some will annihilate, the diagram depicts the stable solution after a sufficiently large time after the rotation.

C. DESCRIPTIONS OF THE VIDEOS

Supplementary Video 1: Skyrmion and domain wall creation in a Pd/Fe/Ir(111) stripe. Real-space dynamics of the skyrmion and DW creation. For the skyrmion the field is applied to two thirds of the width of the stripe. In the case of the DW, the full edge is affected by the field. The lower panel of the animation shows the out-of-plane component of the magnetization along the x-direction. System size: $100 \times 30 \times 1c^3$, $c = 0.233$ nm, with material parameters for Pd/Fe/Ir(111); $\nu = 2$ GHz, $B = 10$ T.

Supplementary Video 2: Continuous skyrmion creation in a Pd/Fe/Ir(111) stripe. Real-space magnetization dynamics steered by a continuously rotating effective field at the left edge of the stripe. The skyrmion density increases until the sample is saturated and no further skyrmions can be induced without destroying previously generated quasiparticles. System size: $100 \times 30 \times 1c^3$, $c = 0.233$ nm, with material parameters for Pd/Fe/Ir(111); $\nu = 2$ GHz, $B = 2.5$ T.

Supplementary Video 3: Creation of a mixed sequence of DWs and skyrmions in a Pd/Fe/Ir(111) stripe. Starting from a field-polarized state, multiple different field operations are performed to generate a mixed sequence of skyrmions and DWs. The only parameter varied to create either a skyrmion or a DW is the field amplitude with $B_{\mathrm{Sk}} = 2.5$ T and $B_{\mathrm{DW}} = 3.5$ T according to (Fig. 1(e)) of the main text. System size: $100 \times 30 \times 1c^3$, $c = 0.233$ nm, with material parameters for Pd/Fe/Ir(111); $\nu = 2$ GHz.

Supplementary Video 4: Skyrmion creation and annihilation in a Pd/Fe/Ir(111) stripe. Starting from a field-polarized state, two writing operations and one deletion with reversed rotational sense of the external field are performed. System size: $60 \times 30 \times 1c^3$, $c = 0.233$ nm, with material parameters for Pd/Fe/Ir(111); $\nu = 2$ GHz, $B = 2.5$ T
